# Exploration of dreams in Charaka Samhita – an Ayurveda text and their content analysis of prodromal dreams in various conditions

**DOI:** 10.1192/j.eurpsy.2024.1728

**Published:** 2024-08-27

**Authors:** A. K. Iyer, W. Upadhyaya

**Affiliations:** ^1^Unaffiliated, Independent, London, United Kingdom; ^2^Centre Ahimsa, Paris, France

## Abstract

**Introduction:**

Research into dreams, have shown the association between increased frequency of distressing dreams, specific content themes (analysed using the Hall Van de Castle system) and greater incidence of progression of neurological conditions and dementia. The history of predicting illnesses by the content of dreams, in the western world is popularly traced backed to the ancient Greek medicine. This stimulates the curiosity if any such practices existed in the ancient medical practises of the eastern world. Ayurveda is one such traditional system of medicine, that is native to the Indian subcontinent. Charaka Samhita is one of the oldest texts on Ayurveda consisting of 8 sections and 120 chapters totally. This text was selected for the purpose of this review, with the line of enquiry such as what does Ayurveda say about dreams associated with illnesses? What are the contents of such dreams? Furthermore, the dream content analysis was done using the Hall Van de Castle system, which is probably the first time being done on an Ayurveda text content.

**Objectives:**

1) To explore if, Charaka Samhita mentions, describes dreams in relation to illnesses, stages of illnesses and their prognosis. 2) To analyse content of the dreams seen in prodromal stage of illnesses.

**Methods:**

1-The Charaka Samhita text was scanned chapter by chapter, to answer the questions- a) What are the types of dreams? b) Are any associated with illnesses? c)Are any dreams mentioned in the prodromal stage of illnesses? d)What do they imply? e)What are their contents? 2- The contents of prodromal dreams were analysed against the categories of Hall Van de Castle system.

**Results:**

As per Charaka Samhita, the types of dreams are, i) those based on what was seen ii) heard iii) reflected upon iv) desired v) imagined vi) those of prophetic type and vii) those caused by illnesses. Specific dreams in the prodromal stage, predict manifestation of specific illnesses (mild or fatal). In the diverse dream contents (18 themes mentioned) ranging from things animals to gods and demons, except the elements of the past, rest of the general categories occur, at least once. The categories characters, objects, activities and social interactions were more common than the rest.

**Conclusions:**

Thus akin to the ancient Greek medicine, Ayurveda too had the practice of predicting illnesses based on the dream contents.

**Disclosure of Interest:**

None Declared

